# Supplementary material for: A novel framework for three-dimensional electrical impedance tomography reconstruction of maize ear via feature reconfiguration and residual networks
Source: PeerJ Comput Sci. 2024 Apr 11;10:e1944. doi: 10.7717/peerj-cs.1944 (PMC11042020; doi:10.7717/peerj-cs.1944)
Supplement: Supplemental Information 2 [file peerj-cs-10-1944-s002.docx]

# Supplementary Information

## Ⅰ. The process of creating a dataset

The simulated datasets were generated using EIDORS 3.10 in conjunction with Netgen 5.3. Upon acceptance of the paper, the code will be made publicly available on GitHub. Below are pertinent details.

### Acrylic simulation dataset

1、Dimensions of the model and electrode parameters

| Parameter Name | Variable Name | Parameter Value |
| --- | --- | --- |
| Model dimensions | cyl_shape | [0.08,0.05,0.004] |
| Number of electrodes per layer | nelec | 16 |
| Electrode ring position | ring_vert_pos | [0.0196, 0.0392, 0.0588] |
| Electrode shape | elec_shape | [0.001,0,0.02] |

2、Key information

(1) Use netgen to create a cylindrical model:

ng_mk_cyl_models(cyl_shape,[nelec,ring_vert_pos],elec_shape)

(2) Establish a general EIT model, set the three-dimensional field structure 'b3cr', and adopt a structure with 3 layers of 16 electrodes:

mk_common_model('b3cr',[16,3])

(3) Create EIDORS excitation and measurement pattern structures, adopt adjacent excitation and adjacent measurement modes, and use the fwd_solve function to obtain the empty field voltage vh:

mk_stim_patterns(nelec,nrings,[0,1],[0,1],{'no_meas_current','rotate_meas'},0.03);

vh = fwd_solve()

(4) To insert the simulated object to be tested, first determine the mathematical expression of the object to be tested. For example, in three-dimensional space, the mathematical expression of a sphere is (x-x0)² + (y-y0)² + (z-z0)² = r²:

Sphere code: strcat('(x-',string(x_d),').^2+(y-',string(y_d),').^2+(z-',string(z_d),').^2

<',string(r),'^2');

Cylindrical code: strcat('(x-',string(x_d),').^2+(y-',string(y_d),').^2<',string(r),'^2');

Rectangular code: strcat('abs(x-',string(x_d),')+abs(y-',string(y_d),')<',string(r));

(5) Add the simulated object to be tested to the three-dimensional field, and finally obtain the structure img2 containing the object to be tested:

select_fcn = inline(expression,'x','y','z');

memb_frac = elem_select(img1.fwd_model, select_fcn);

img2 = mk_image(img1, memb_frac*cond );

(6) Use the fwd_solve function to output the boundary voltage containing the object to be tested, which is the boundary voltage without noise:

vi=fwd_solve(img2);

(7) Use the add_noise function, set the snr parameter to add noise with different signal-to-noise ratios,

for example, snr = 30/40/50:

add_noise(10^(snr/20) ,vi);

### Corn ear model simulation dataset

1. Parameters of the test domain and electrodes.

| Parameter Name | Variable Name | Parameter Value |
| --- | --- | --- |
| Model dimensions | cyl_shape | [0.066,0.0355,0.003] |
| Number of electrodes per layer | nelec | 16 |
| Electrode ring position | ring_vert_pos | [0.011, 0.033, 0.055] |
| Electrode shape | elec_shape | [0.001,0,0.02] |

2. Key Information

(1) **Parameter Definition**:

- `base_scale_x/y/z`: These parameters define the attenuation speed range in each direction. The attenuation speed determines how conductivity decreases as distance increases.

- `diffusion_range`: This is a factor used to randomly adjust the attenuation speed, giving the conductivity distribution in the model some randomness.

- `center_position_perturbation`: Defines the range of random perturbation for the center position, increasing the uncertainty of the center position.

- `distance_perturbation_scale`: Adds random perturbation to the calculated distances, so that each element has a slightly different distance to the center.

- `min/max_conductivity_range`: Defines the minimum and maximum range for conductivity.

- `num_centers_z_range/num_centers_xy_range`: Defines the number of centers in the Z direction and in the XY direction.

(2) **Calculate the Center Coordinates of All Elements**:

- Using the node and element information of the model, calculate the center coordinates for each element.

(3) **Determine the Boundaries of the 3D Field**:

- Find the minimum and maximum coordinates of all element centers in the model.

(4) **Randomly Select Centers in the Z Direction**:

- Randomly select centers in the Z direction.

(5) **Initialize Conductivity**:

- Initialize a conductivity value for each element, starting at zero.

(6) **For Each Center in the Z Direction**:

- Enter a loop and operate on each center in the Z direction.

(7) **Select Centers in the XY Direction**:

- For each center in the Z direction, enter another loop to select centers in the XY direction.

(8) **Calculate Distance Between Element and Center**:

- Calculate the distance from the center of each element to the current center in both XY and Z directions.

(9) **Determine Diffusion Speed**:

- Randomly select an attenuation speed within the specified range and consider random perturbation using `diffusion_range`.

(10) **Calculate the Effect on Conductivity**:

- Use an exponential function to simulate how conductivity varies with distance. This uses both the attenuation speed and the distance between the element and the center.

(11) **Normalize and Map Conductivity**:

- Use a normalization method to map the range of conductivity to the specified minimum and maximum conductivity range.

(12) **Output Results**:

- Assign the calculated conductivity distribution to `img1.elem_data`.

## Ⅱ. Simulation experiment imaging results

Table 1. Evaluation metrics of the simulation

experiment under the ideal noise-free condition.

|  | | Object 1 | Object 2 | Object 3 | Object 4 |
| --- | --- | --- | --- | --- | --- |
| None | RMSE | 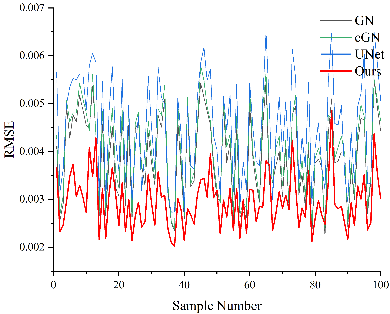 | 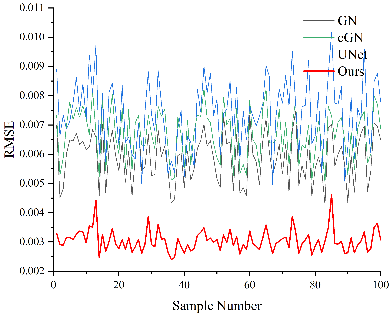 | 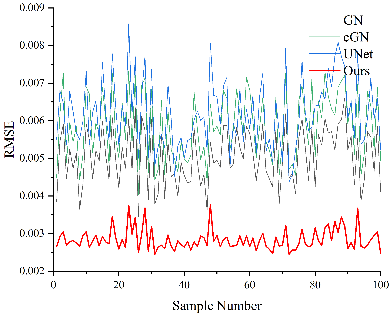 | 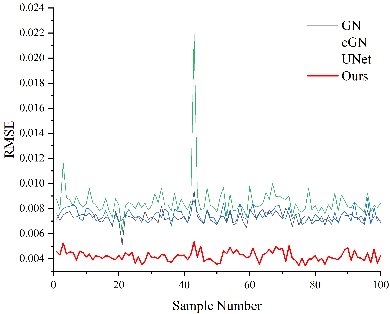 |
|  | CC | 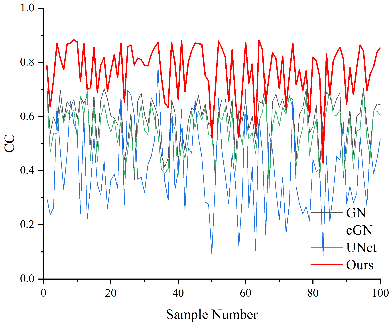 | 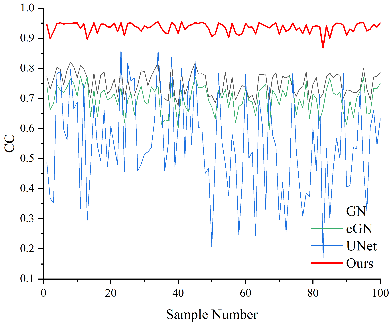 | 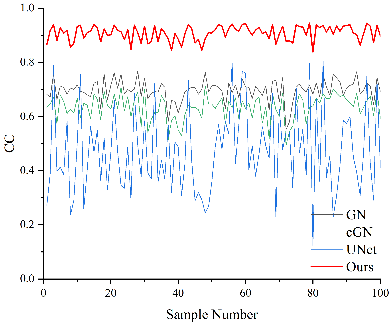 | 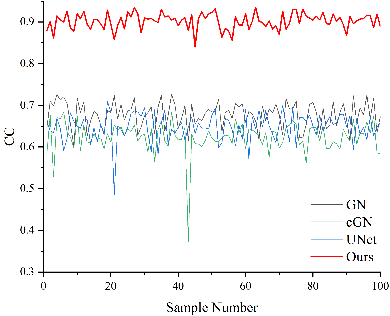 |
|  | SSIM | 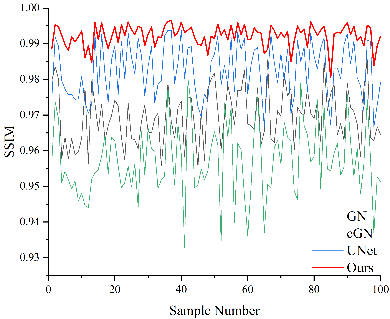 | 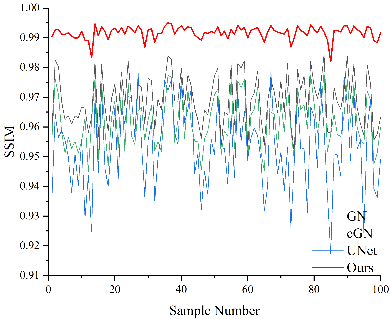 | 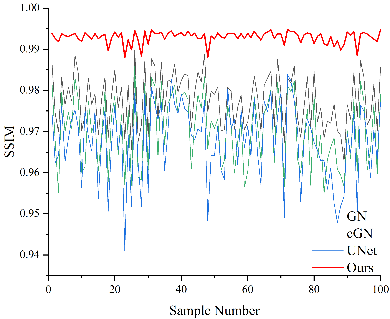 | 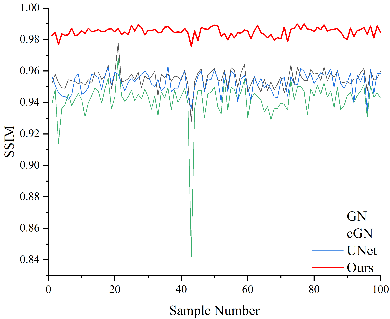 |

Table 2. Evaluation metrics of the simulation

experiment with a signal-to-noise ratio of 50dB.

|  | | Object 1 | Object 2 | Object 3 | Object 4 |
| --- | --- | --- | --- | --- | --- |
| 50dB | RMSE | 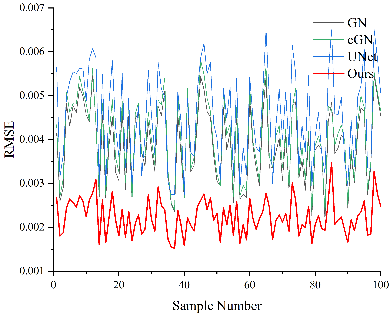 | 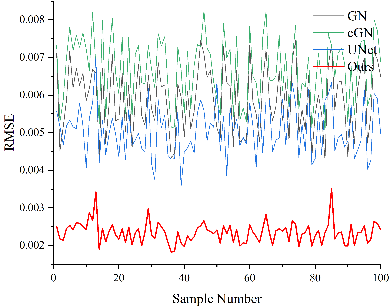 | 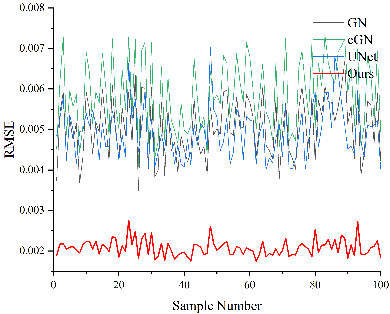 | 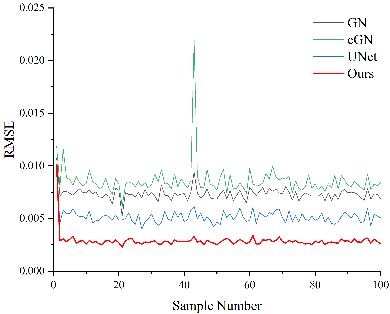 |
|  | CC | 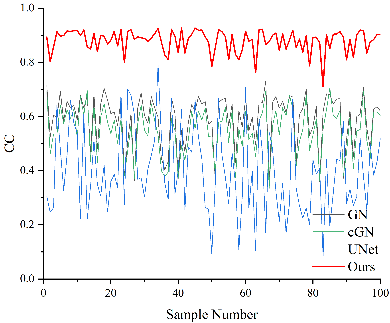 | 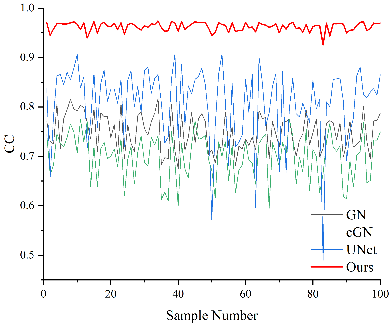 | 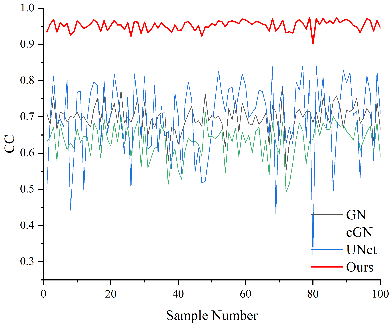 | 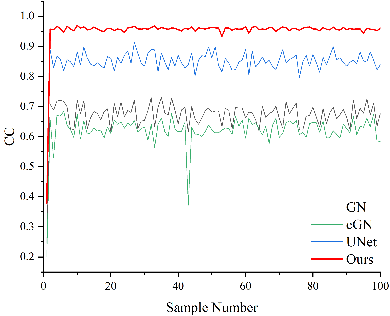 |
|  | SSIM | 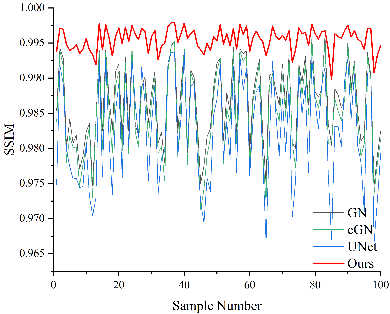 | 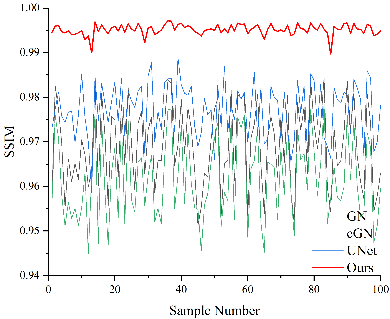 | 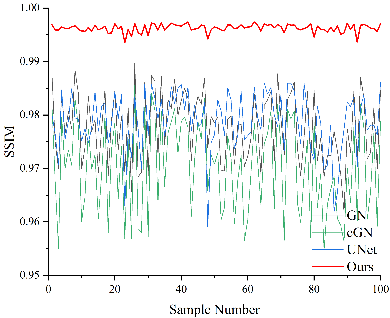 | 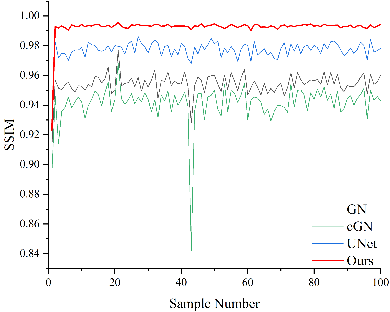 |

Table 3. Evaluation metrics of the simulation

experiment with a signal-to-noise ratio of 40dB.

|  | | Object 1 | Object 2 | Object 3 | Object 4 |
| --- | --- | --- | --- | --- | --- |
| 40dB | RMSE | 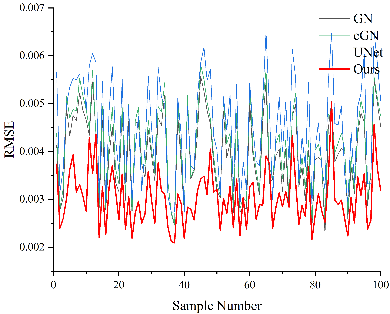 | 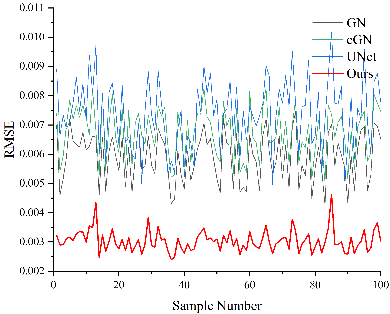 | 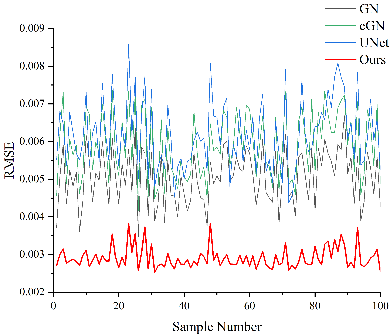 | 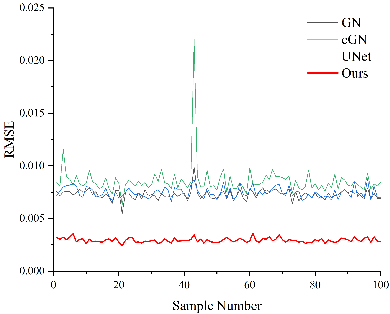 |
|  | CC | 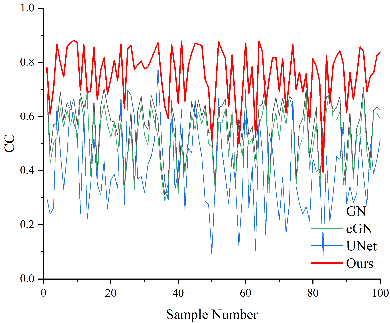 | 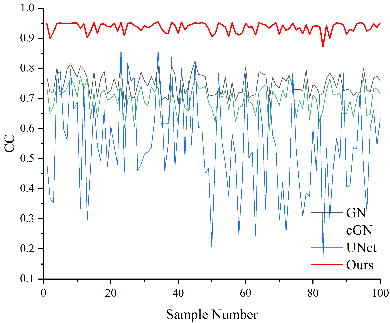 | 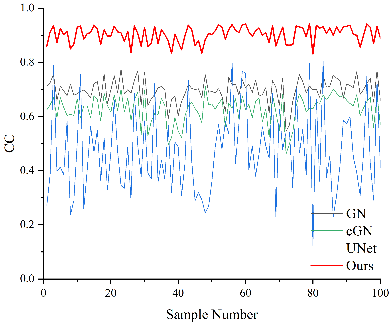 | 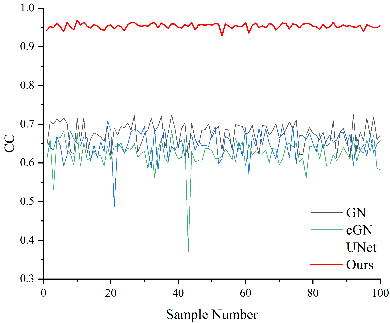 |
|  | SSIM | 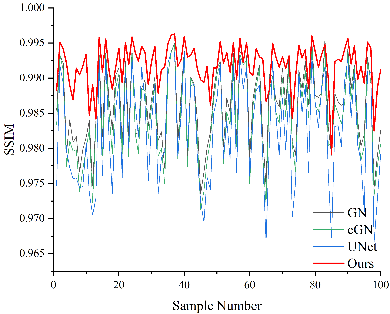 | 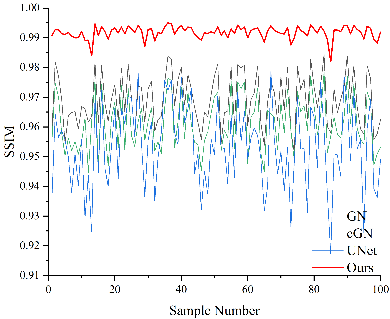 | 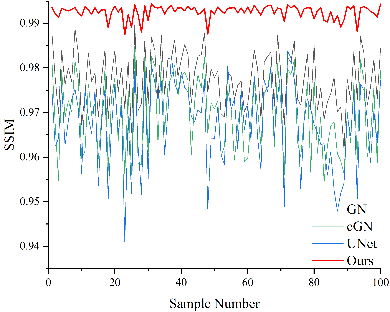 | 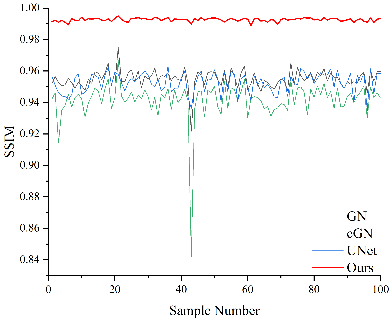 |

Table 4. Evaluation metrics of the simulation

experiment with a signal-to-noise ratio of 30dB.

|  | | Object 1 | Object 2 | Object 3 | Object 4 |
| --- | --- | --- | --- | --- | --- |
| 30dB | RMSE | 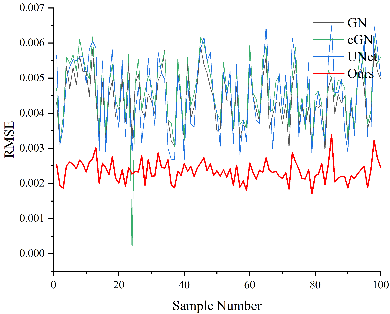 | 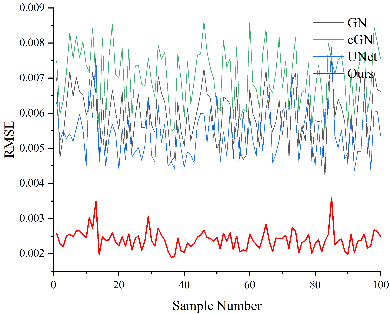 | 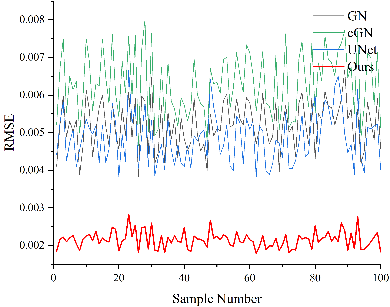 | 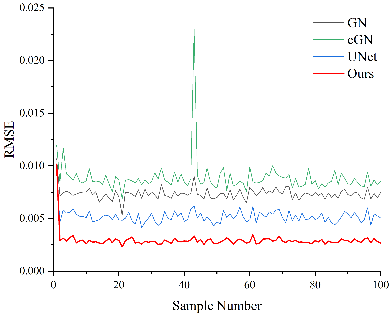 |
|  | CC | 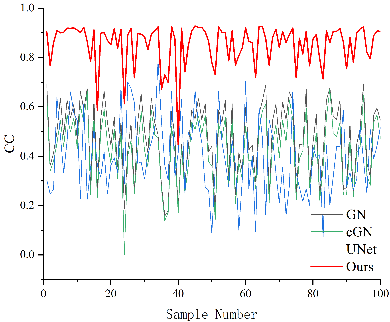 | 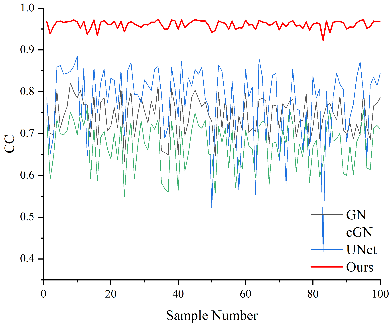 | 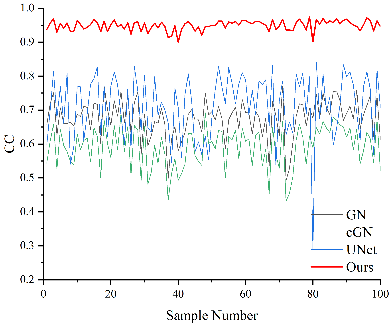 | 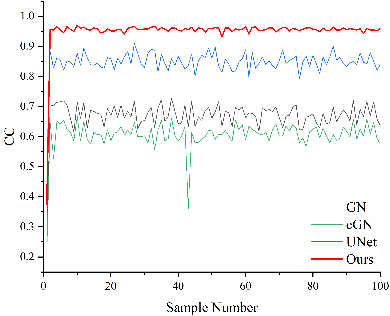 |
|  | SSIM | 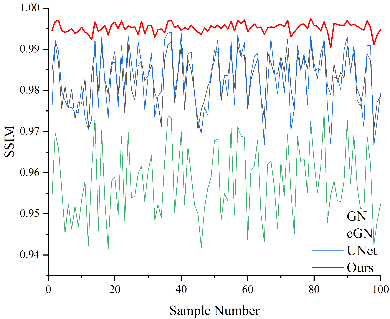 | 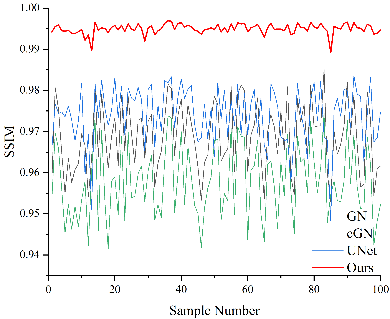 | 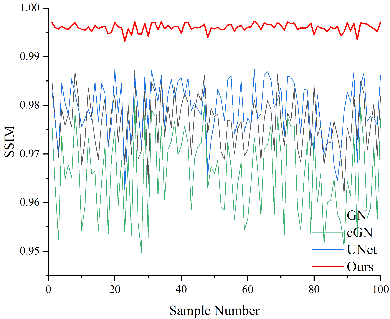 | 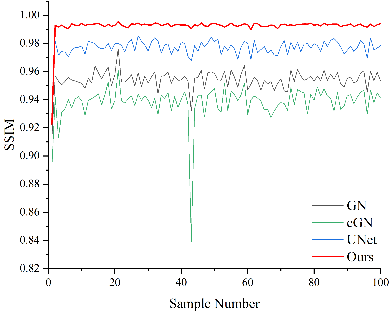 |

Table 5. Evaluation metrics of the simulation

experiment with a signal-to-noise ratio of 20dB.

|  | | Object 1 | Object 2 | Object 3 | Object 4 |
| --- | --- | --- | --- | --- | --- |
| 20dB | RMSE | 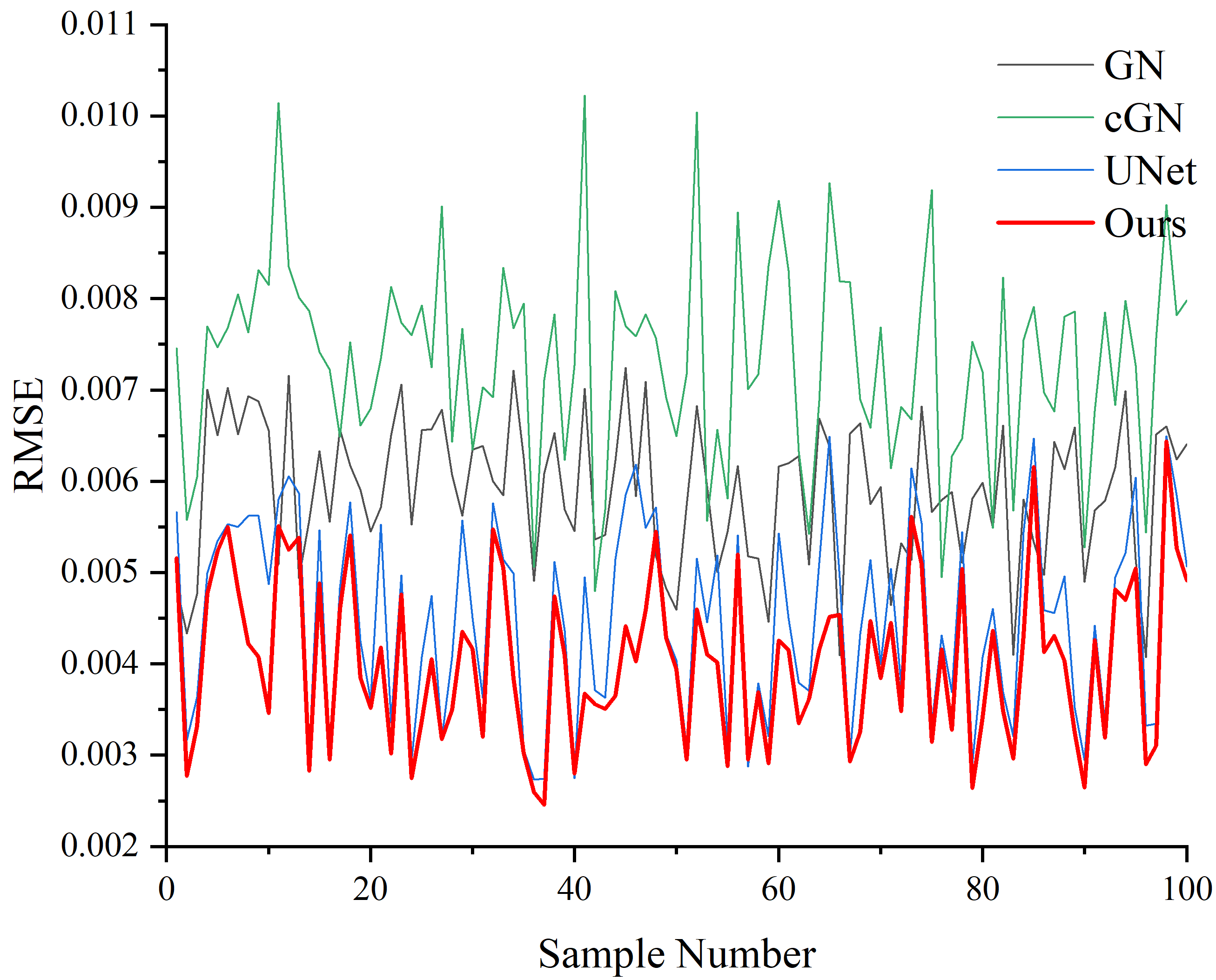 | 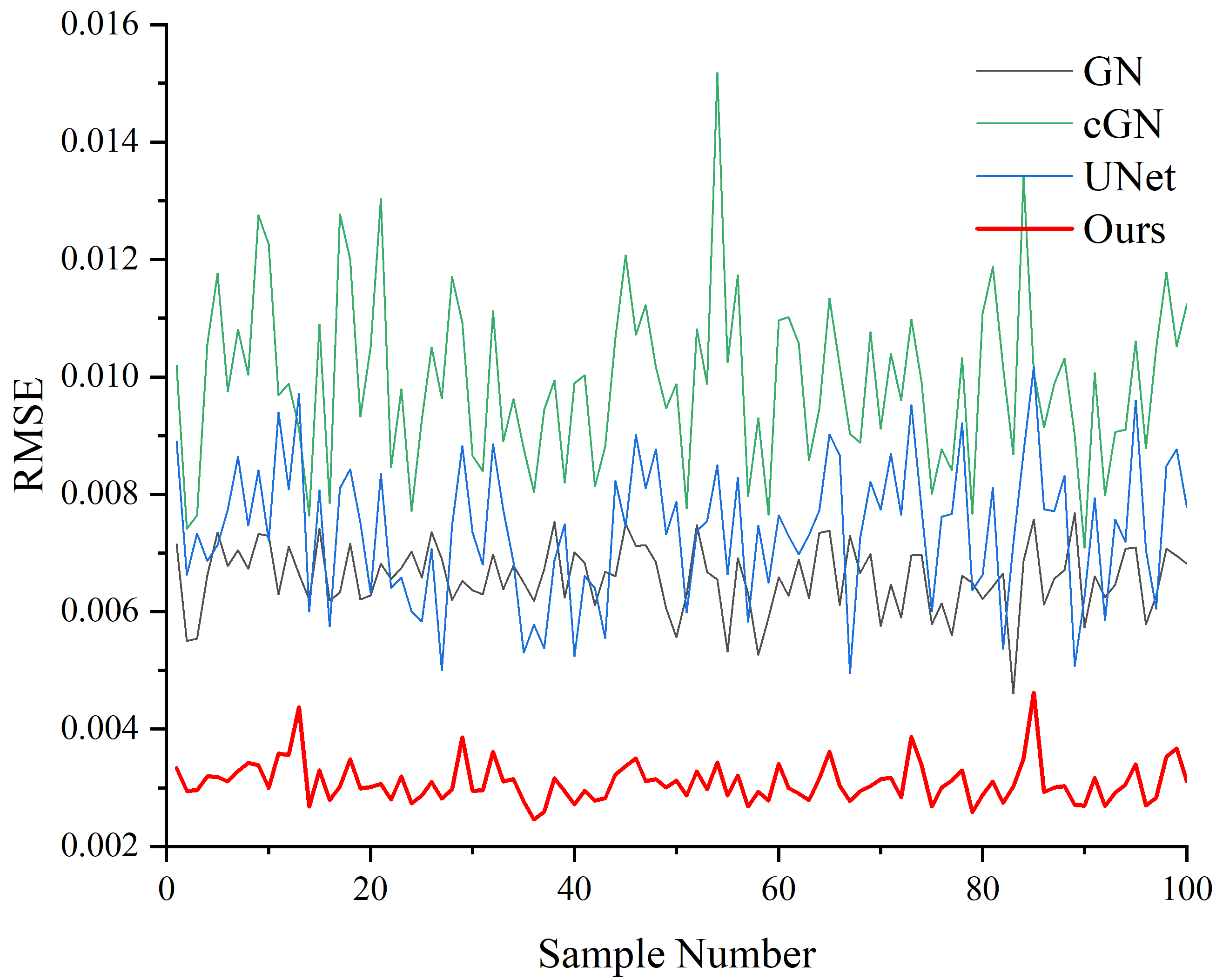 | 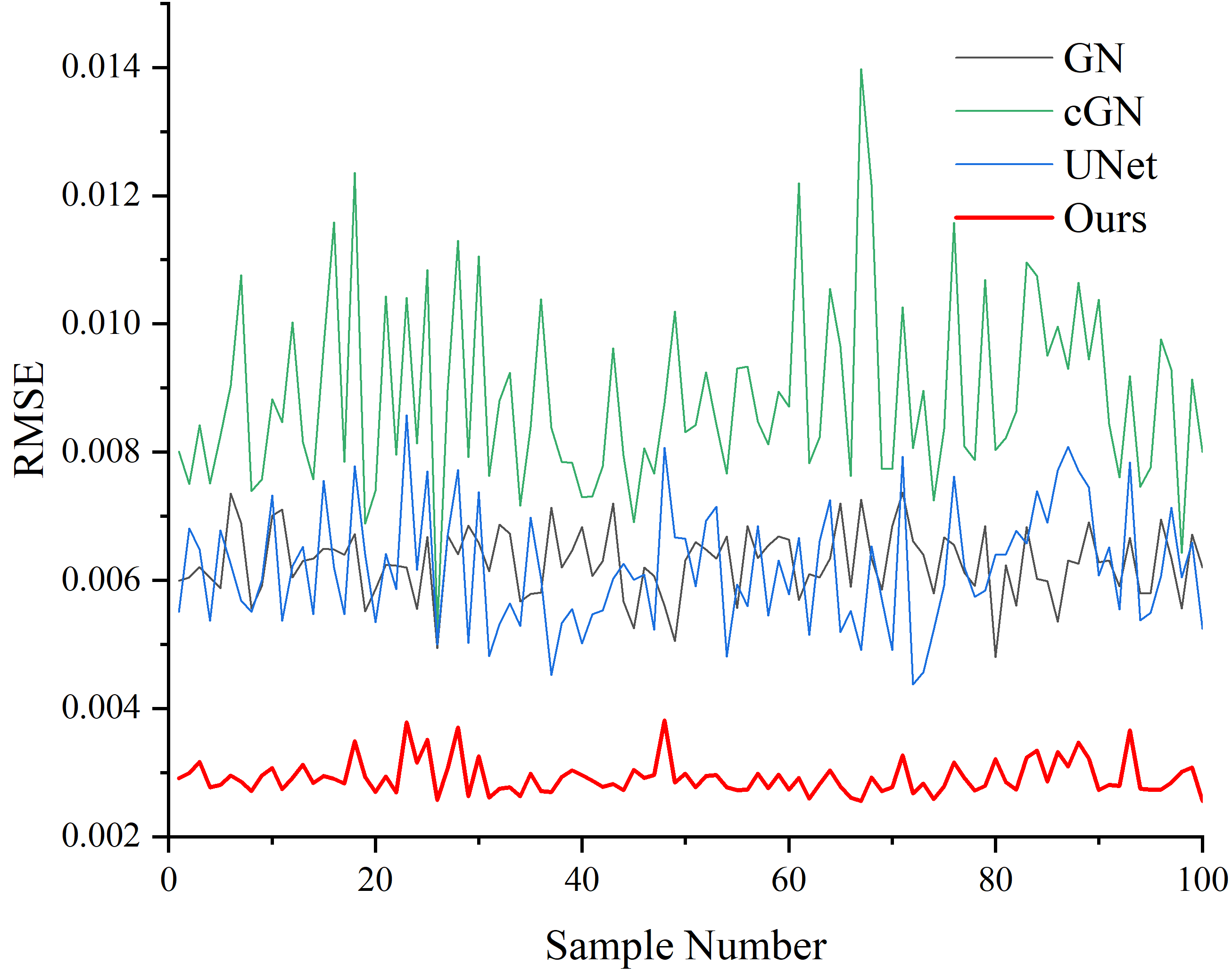 | 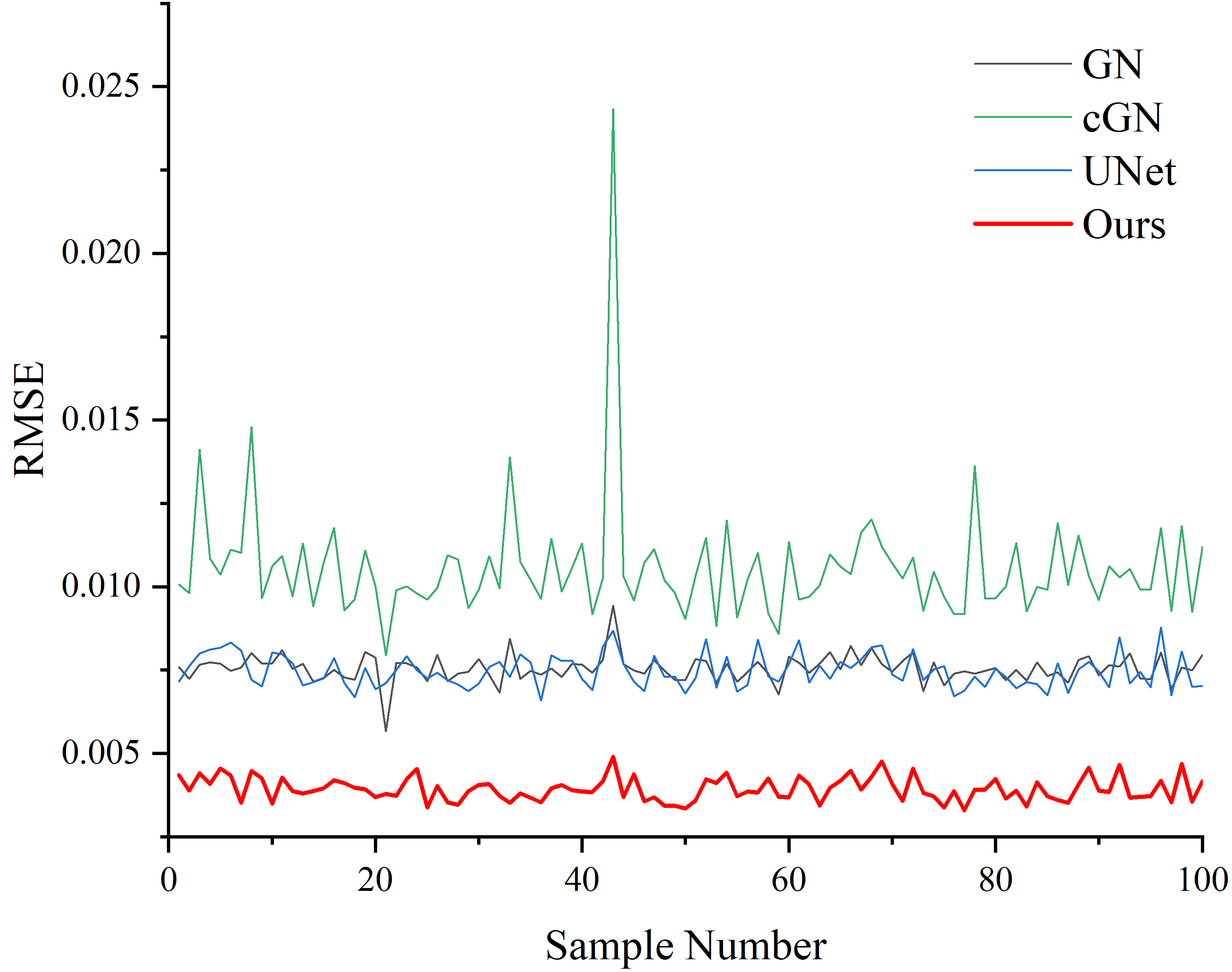 |
|  | CC | 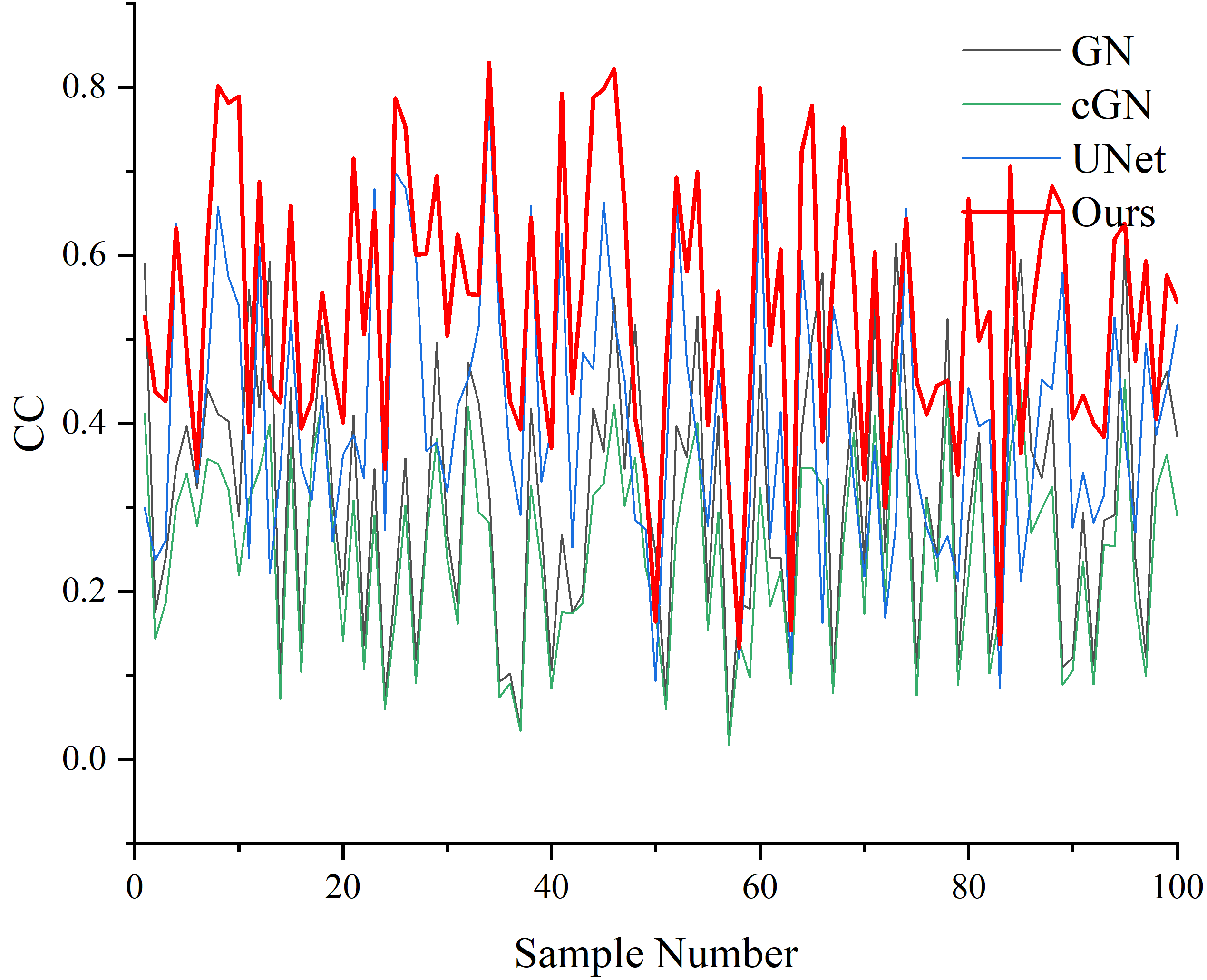 | 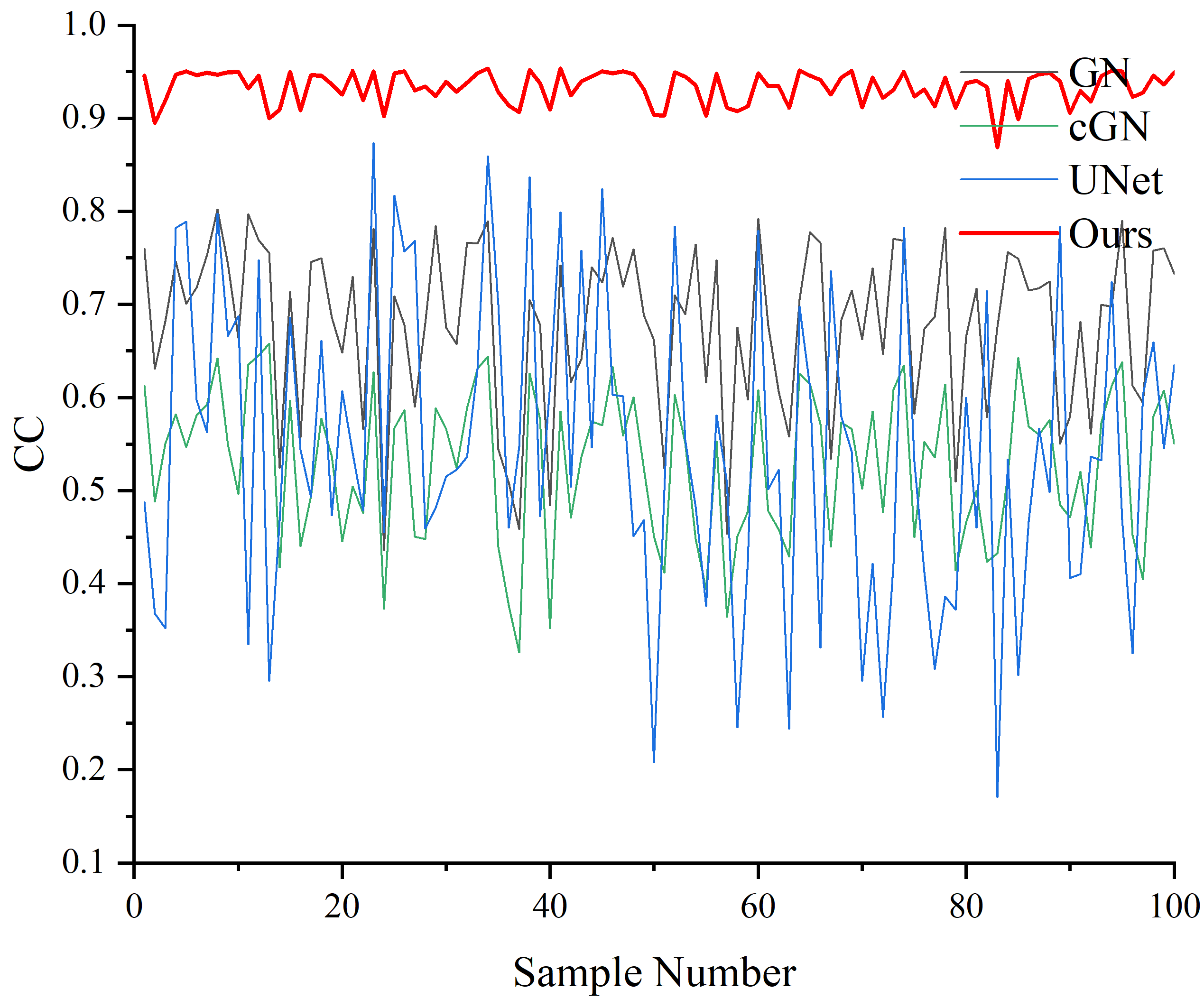 | 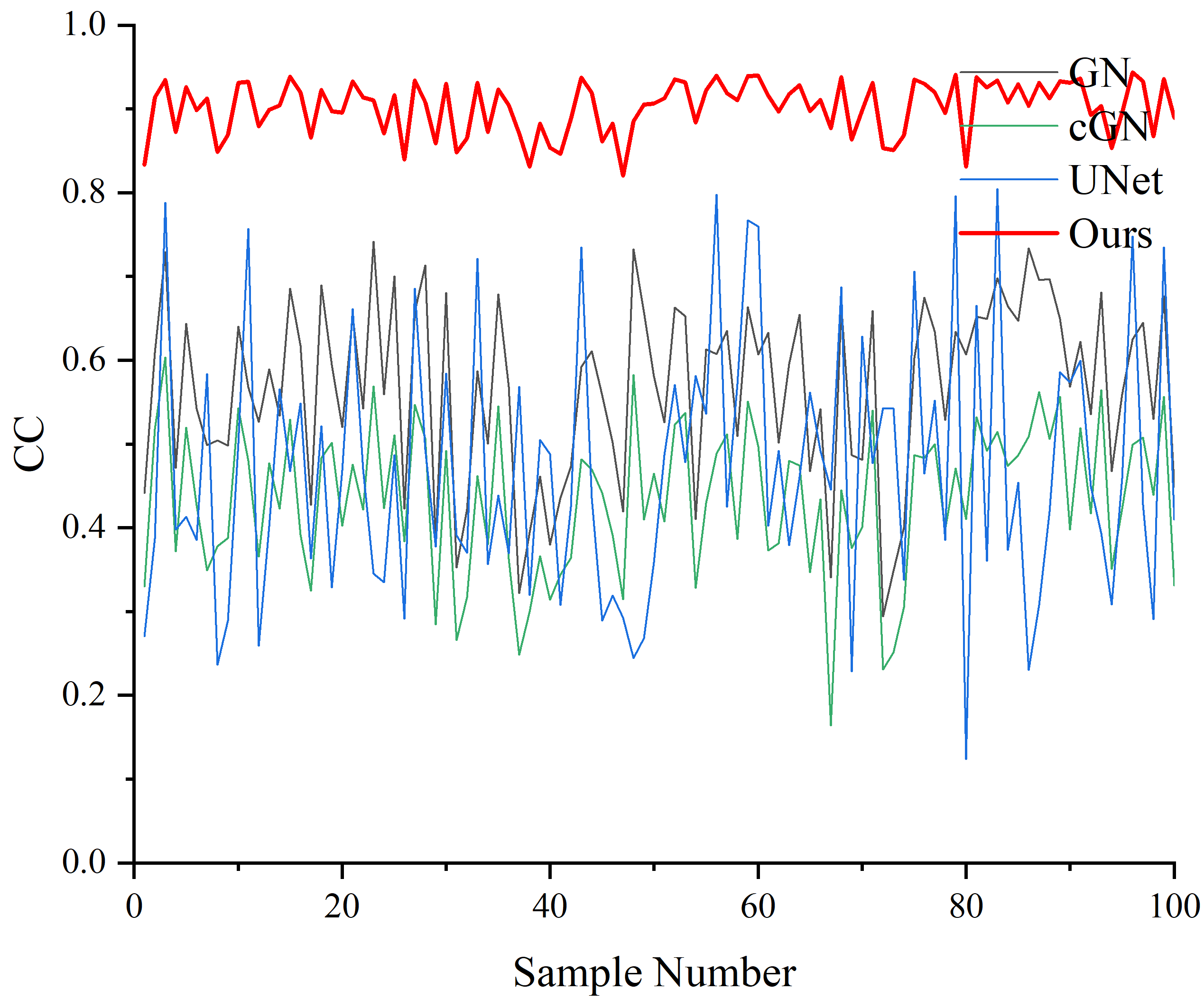 | 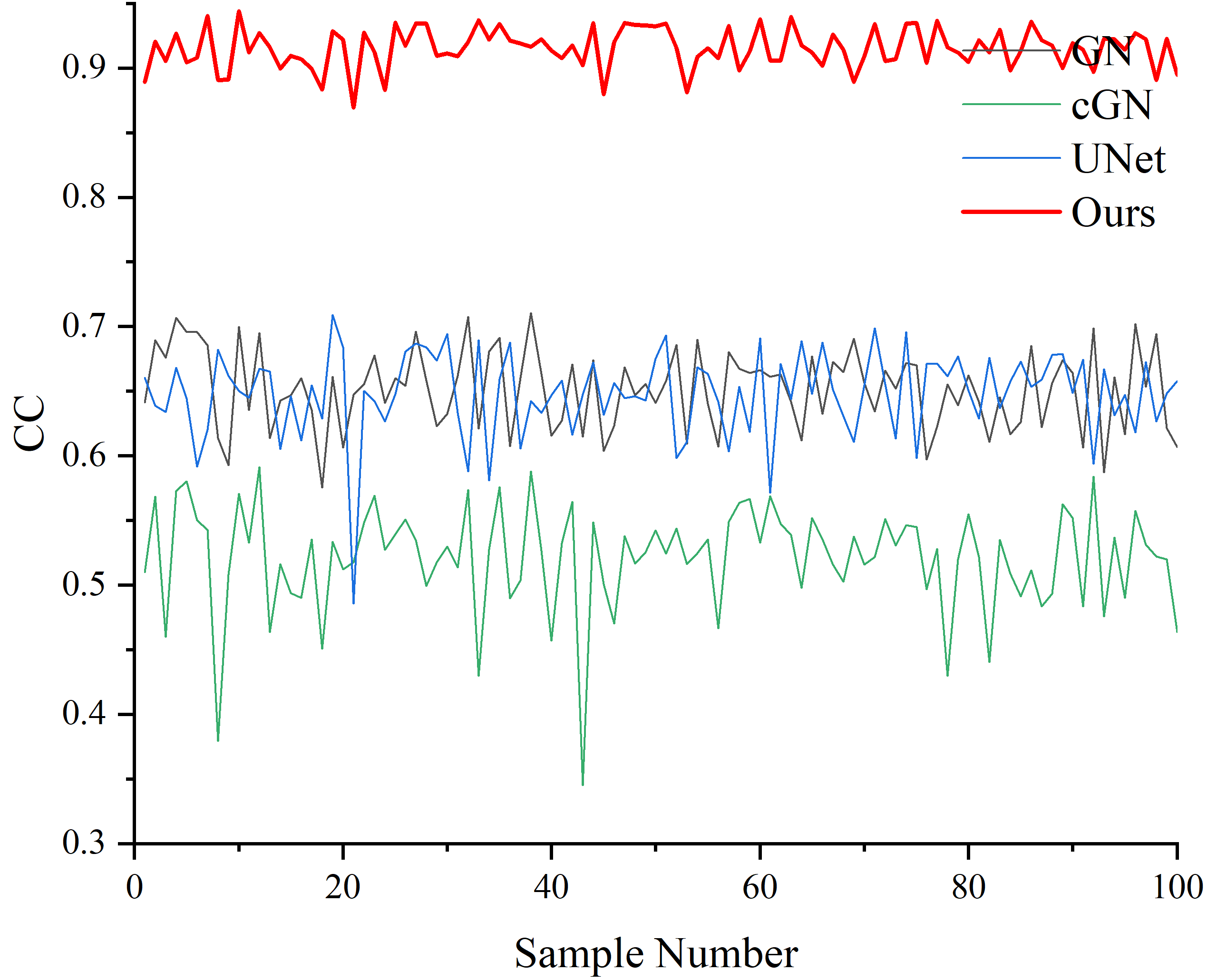 |
|  | SSIM | 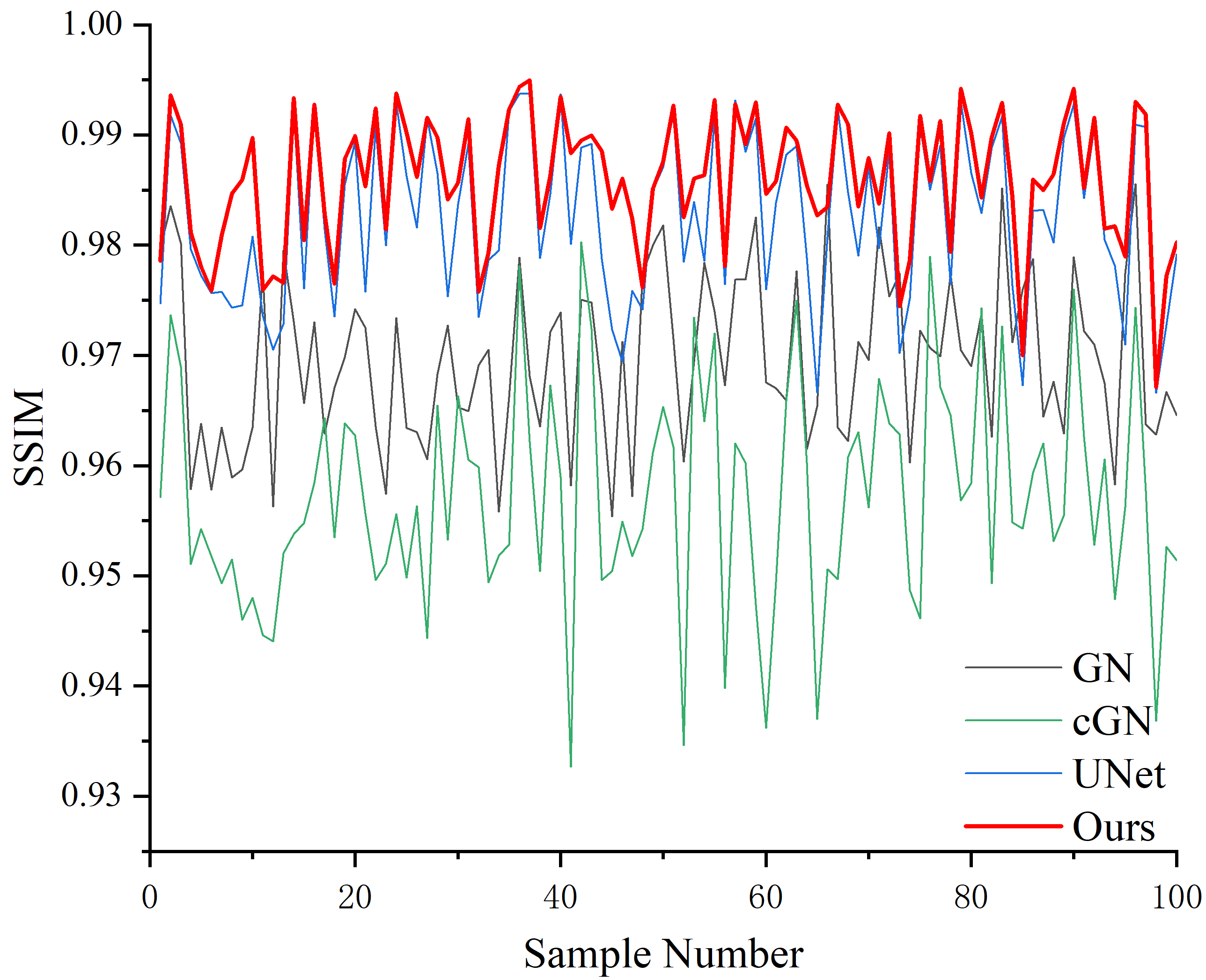 | 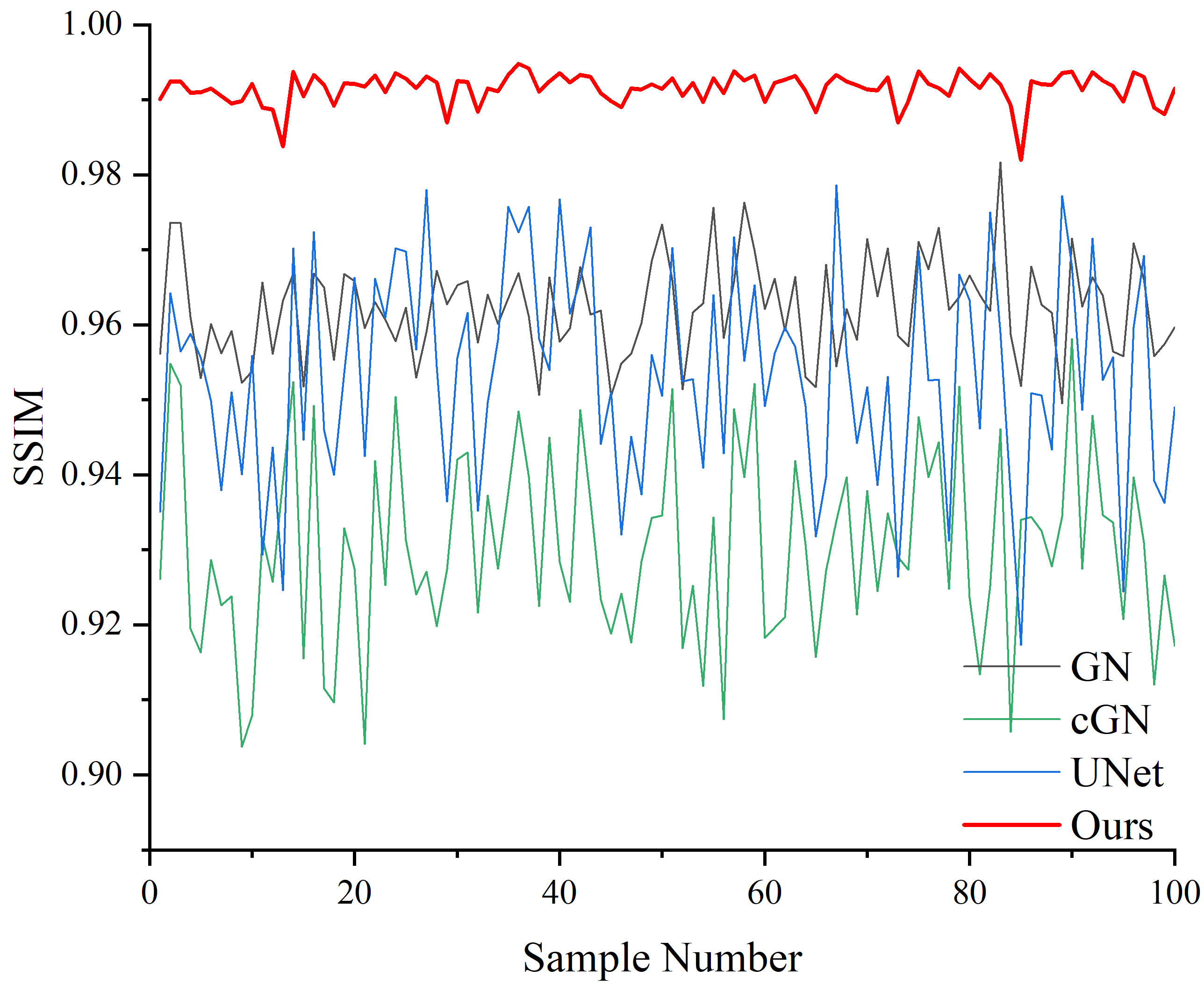 | 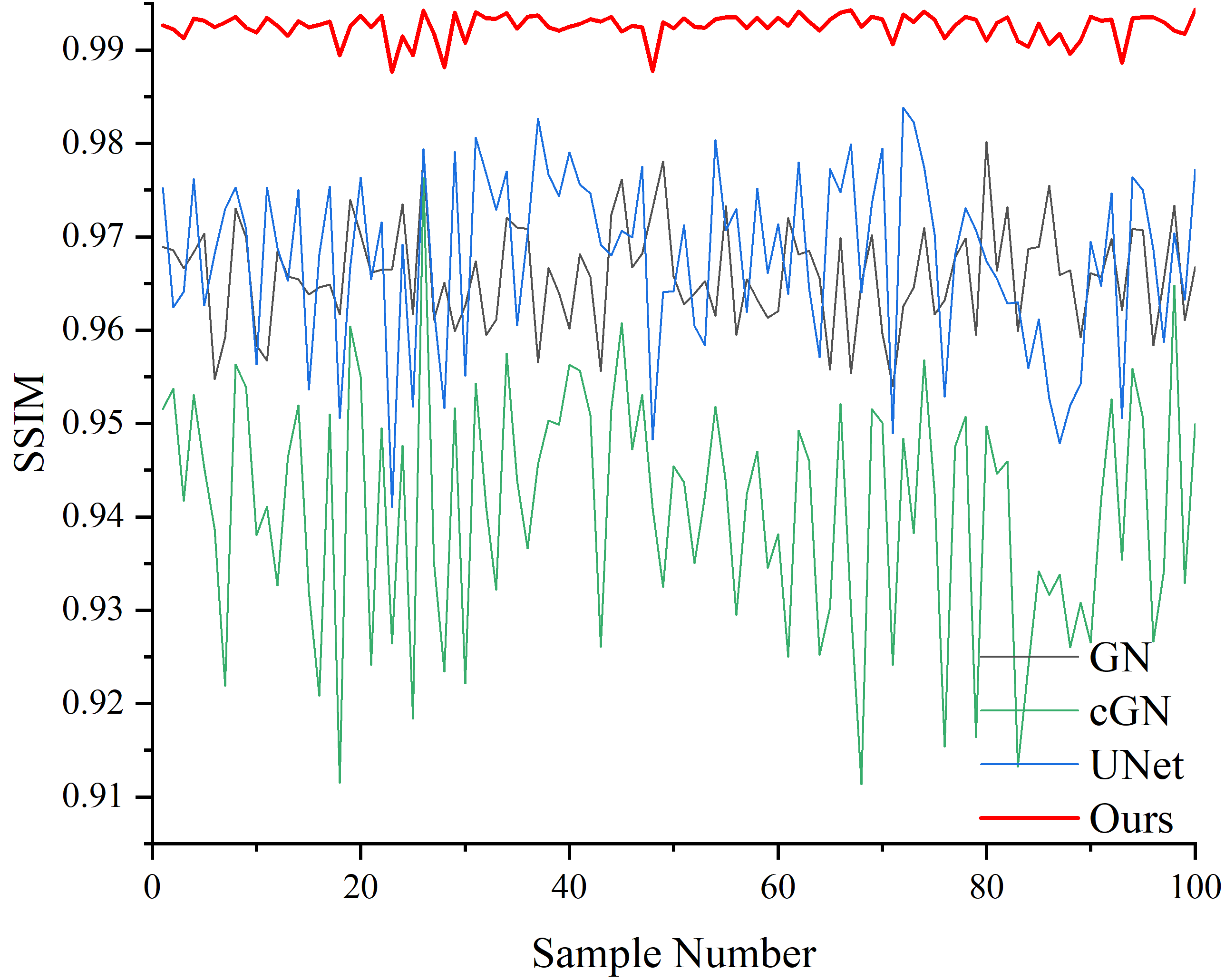 | 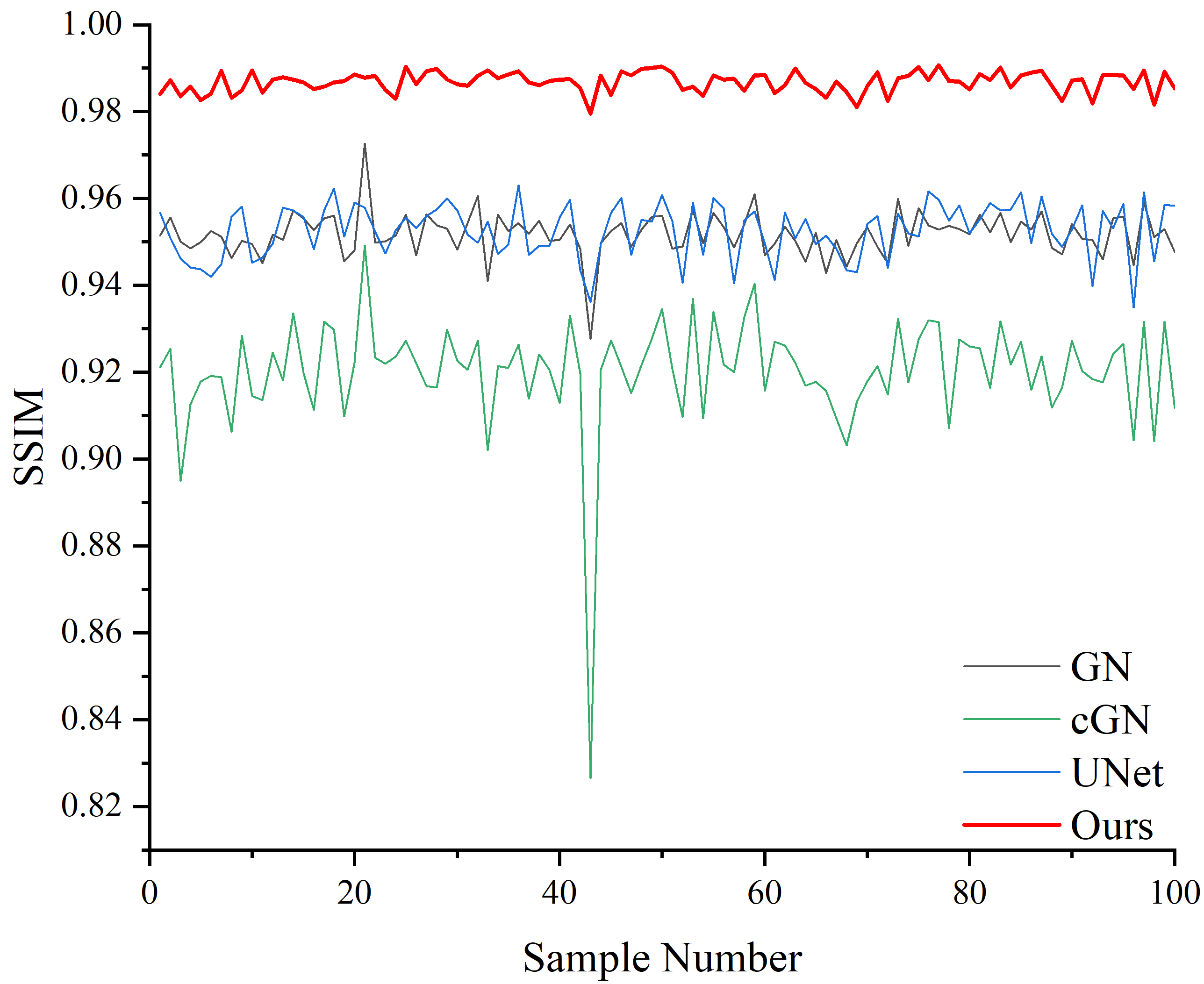 |

## Ⅲ. Confidence interval of the experimental results for Object4.

100 samples, using Z-score to calculate the 95% confidence

| Table 6. Confidence interval under the ideal noise-free condition. | | | |
| --- | --- | --- | --- |
|  | RMSE | CC | SSIM |
| GN | [0.7202, 0.7398]_*0.01_ | [0.6717,0.6837] | [0.9539, 0.9561] |
| cGN | [0.825, 0.880] _*0.01_ | [0.6163, 0.6311] | [0.9394, 0.9442] |
| UNet | [0.737, 0.757] _*0.01_ | [0.6417, 0.6547] | [0.9514, 0.9538] |
| Ours | [0.412, 0.428] _*0.01_ | [0.8982, 0.9056] | [0.9844, 0.9855] |

| Table 7. Confidence interval at SNR=50dB. | | | |
| --- | --- | --- | --- |
|  | RMSE | CC | SSIM |
| GN | [0.821, 0.879] _*0.01_ | [0.6621,0.6821] | [0.9529, 0.9557] |
| cGN | [0.829, 0.891] _*0.01_ | [0.6092, 0.6312] | [0.9388, 0.9440] |
| UNet | [0.504, 0.528] _*0.01_ | [0.8378, 0.8578] | [0.976, 0.9784] |
| Ours | [0.274, 0.306] _*0.01_ | [0.941, 0.963] | [0.9911, 0.9939] |

| Table 8. Confidence interval at SNR=40dB. | | | |
| --- | --- | --- | --- |
|  | RMSE | CC | SSIM |
| GN | [0.710,0.730] _*0.01_ | [0.6706,0.6822] | [0.9538, 0.9560] |
| cGN | [0.826, 0.884] _*0.01_ | [0.6161, 0.6305] | [0.9392, 0.9440] |
| UNet | [0.740, 0.760] _*0.01_ | [0.6417, 0.6547] | [0.9514, 0.9538] |
| Ours | [0.29, 0.3] _*0.01_ | [0.9523, 0.9549] | [0.9924, 0.9928] |

| Table 9. Confidence interval at SNR=30dB. | | | |
| --- | --- | --- | --- |
|  | RMSE | CC | SSIM |
| GN | [0.718,0.742] _*0.01_ | [0.6597, 0.6798] | [0.9528, 0.9556] |
| cGN | [0.853, 0.915] _*0.01_ | [0.5945, 0.6145] | [0.9356, 0.9406] |
| UNet | [0.508, 0.532] _*0.01_ | [0.836, 0.856] | [0.9757, 0.9781] |
| Ours | [0.274, 0.306] _*0.01_ | [0.9394, 0.9614] | [0.9909, 0.9937] |
